# Risk Analysis of Viral Diseases in Infected Pig Farms during the Lockdown Period in China, January to May 2020

**DOI:** 10.3390/ijerph19063215

**Published:** 2022-03-09

**Authors:** Jieru Wang, Xiaojie Zhu, Chang Cai, Xiaocheng Pan, Chonglong Wang

**Affiliations:** 1 Anhui Province Key Laboratory of Livestock and Poultry Product Safety Engineering, Livestock and Poultry Epidemic Diseases Research Center of Anhui Province, Key Laboratory of Pig Molecular Quantitative Genetics of Anhui Academy of Agricultural Sciences, Institute of Animal Husbandry and Veterinary Science, Anhui Academy of Agricultural Sciences, Hefei 230031, China; wangjr0317@163.com; 2 The National Key Laboratory of Agricultural Microbiology, Huazhong Agricultural University, Wuhan 430070, China; xiaojie.zhu@murdoch.edu.au; 3 China-Australian Joint Laboratory for Animal Health Big Data Analytics, College of Animal Science and Technology & College of Veterinary Medicine, Zhejiang A&F University, Hangzhou 311300, China; c.cai@murdoch.edu.au

**Keywords:** biosecurity, intensive pig farms, risk analysis, viral disease, lockdown period, practice

## Abstract

Biosecurity plays a critical role in preventing and controlling the introduction and spread of infectious diseases. The COVID-19 pandemic in China triggered a nationwide lockdown policy which reduced most of the daily activities of people, but the pig industry was encouraged to ensure the pork supply. An investigation of biosecurity practices in intensive pig farms across several provinces in China was conducted in June 2020 via questionnaire to evaluate the factors that may pose viral diseases risk to the farms during the lockdown period from January to May 2020. A total of 50 farms in 12 provinces of China were engaged. Fourteen of them were classified as positive farms since at least one viral disease was presented during this period, including porcine reproductive and respiratory syndrome (seven farms), porcine epidemic diarrhea (three farms), and pseudorabies (one farm). The other three farms only reported their disease positive status but refused to release disease names. The overall farm level prevalence of viral disease was 28.0% (95%CI: 16.3–42.5%). A logistic regression model was built to identify risk/protective factors for farm positivity. In the multivariable logistic regression model, the risk factor of dead pig ‘removal by the others’ (OR = 8.0, 95%CI: 1.5, 43.5) was found to be significantly associated with viral disease positivity. On-farm incineration pits are highly recommended to be the administered for the harmless treatment of dead pigs. This is not only crucial for controlling the transmission of viral diseases but also plays a key role in reducing activity in the illegal dead meat business. According to previous studies, factors such as adapting an all-in-all-out system, on-farm incineration pits, and requiring workers to wash their hands regularly would reduce the risk of virus transmission, even though these factors did not show significance in our study. The results of our study could help to design better surveillance strategies in China and other countries.

## 1. Introduction

Biosecurity plays a critical role in preventing and controlling the introduction and the spread of infectious diseases. Implementation of biosecurity measures is the frontline of defenses against pathogens [1] and is also crucial in controlling diseases, such as infectious diarrhea [2] and respiratory disorders [3]. However, due to the lack of high-level education of farmers, on-farm biosecurity practices and awareness of biosecurity in Chinese pig farms were very poor [4]. In November 2018, African swine fever (ASF) attacked China [5] and almost all the outbreaks occurred in small and free-range farms in the early stage [6,7]. Scientists believed this was due to their poor biosecurity awareness and inadequate disinfection practices [8,9]. In contrast, large-scale pig farms with strict epidemic prevention systems were less susceptible to this fulminating infectious disease. Therefore, intensive farms tend to survive in the ASF outbreak [10,11]. In addition, due to the pollution caused by small pig farms, the Chinese government is now encouraging larger, more standardized, and vertically integrated production systems [12].

In late 2019, a novel coronavirus (COVID-19) attacked China and subsequently became a pandemic [13]. To control the COVID-19 outbreak, from January to May 2020 the Chinese government implemented a series of measures, such as city closures, traffic control, travel restrictions, and delayed resumption of work to reduce the non-essential activities [14]. In the meantime, the Chinese government made clear the requirement to speed up the resumption of pig production to ensure the people’s daily pork consumption during the lockdown period [15]. Therefore, January to May 2020 was a non-intervention period for the pig production systems in China with only essential activities allowed. We conducted a nationwide biosecurity survey via questionnaire to investigate the viral diseases situation in Chinese intensive pig farms and risk factors associated with it during the lockdown period. Scientific disease prevention and control suggestions will be put forward to help intensive pig farms improving biosecurity operations.

## 2. Materials and Methods

### 2.1. Target Farms and Questionnaire Design

The conducting institute of this study, Anhui Academy of Agricultural Sciences, has cooperation agreements with 55 intensive pig farms in 15 provinces of China, and they are also the sampling targets of this study. Questionnaires together with consent forms were sent to the farm owners to seek their cooperation and participation. If they wished to engage in our study, they were required to share their farm disease records from January to May 2020 by the end of June 2020 and return a signed consent form together with a questionnaire.

We focused our questionnaire survey on three aspects, characteristics of farms, practices and activities that have potential risks to the farms, and requirements for workers. Characteristics of farms included questions on the year of establishment, farm size, number of breeds raised on the farm, and geographical characteristics. In the year of establishment, we paid special attention to the 2018 ASF outbreak in China and hoped to investigate the prevalence difference between old (established before ASF outbreak) and new (established after ASF outbreak) pig farms. For the geographical characteristics, we asked if the farm was near a mountain/hill or highway since both can pose infectious risk to the farm through wild animals or transportations.

Regarding the biosecurity-related practices and activities, we asked if new pigs need to be tested against infectious diseases before being introduced into the farm (testing before introducing new pigs), if the farm bought and sold pigs from Jan to May 2020 (purchasing and selling activities), if a house level all-in-all-out strategy was implemented in the farm, if vehicles were required to be disinfected before entering the farm, and the method for dead pig disposal. There are two main ways of dead pig disposal in commercial pig farms in China, paying people to collect and dispose dead pigs (removal by others), and having an incineration pit for on-farm harmless treatment (on-farm incineration pit). Requirements for workers were if they needed to change their coat and boots and wash their hands before entering the working area.

A detailed description of our questionnaire survey is listed in Table 1. This study was approved by Anhui Academy of Agricultural Sciences, China (Reference number AAAS2020-12).

### 2.2. Disease Definition

To understand the relationship between biosecurity practices and viral diseases, viral disease status on the farm was confirmed via questions: “Have any animals on your farm ever tested positive for any viral diseases between 1 January 2020 to the 31 May 2020?", with the possible answers of yes, no, or not sure; “If yes, which diseases? Which method was used to test your pigs for those diseases? Please specify the method.” Farms with at least one viral disease recorded from 1 January 2020 to 31 May 2020 were considered viral disease positive farms in our study.

### 2.3. Risk Analysis

Disease records and questionnaire information were extracted, stored, and sorted in Microsoft^®^ Excel Version 2005.

The names of diseases were recorded with the farm managers’ permission. Questionnaires with more than 20.0% un-answered questions were considered invalid, and the farms providing the invalid questionnaires were then removed from this study. Answers extracted from valid questionnaires were categorized as binary (such as yes or no) or multi-classification variables. Descriptive analysis was carried out using counts and percentages with a 95% confidence interval (CI), followed by univariable analyses via Chi-square tests. Variables with *p* < 0.20 were kept and entered into a multivariable logistic model using the backward stepwise method. Variables with adjusted *p* < 0.05 were considered as factors that significantly associated with viral disease positive farms during the COVID-19 lockdown period in China 2020. Odds ratios were also calculated in both univariable and multivariable analyses to quantify the risks of each variable. Goodness of fit for the logistic regression model was tested via the Hosmer–Lemeshow test. Discrimination ability of the logistic regression model was determined by the area under curve (AUC) of the receiver operating characteristic (ROC).

## 3. Results

### 3.1. Descriptive Analysis

**Figure 1:**
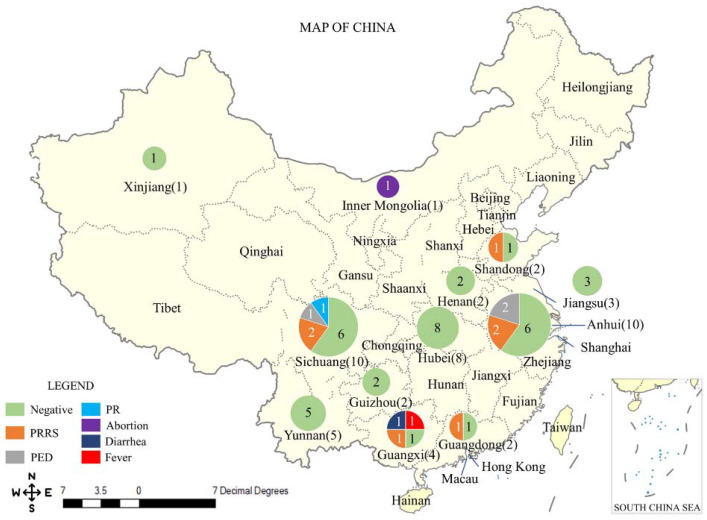
Geographical distribution of farms in the survey and their viral diseases status. The size of the circular chart and the number next to the province name both indicate the number of valid farms engaged in this survey. The colors on the circular charts represent the disease status of the farms, and the numbers represent how many farms have the specific disease status.

Out of 55 cooperative farms, 53 farms from 12 provinces (out of 31 mainland provinces) responded to us. However, three of them provided invalid questionnaires, and they were removed from this study. Hence, 50 farms were included in our final analysis. Anhui and Sichuan provinces provided ten farms, followed by Hubei with eight farms. Five farms are located in Yunnan, four farms located in Guangxi, and others are located in Jiangsu (3), Guangzhou (2), Guizhou (2), Henan (2), and Shandong (2). Xinjiang and Inner Mongolia provinces provided one farm each (Figure 1). These farms were established from 2001 to 2020 of which 11 were newly established after the ASF outbreak.

There were three (6%) commercial grow-to-finish swine farms, 21 (42%) commercial farrow-to-finish swine farms, 19 (38%) breeding swine farms, and seven (14%) wean-to-finish swine farms in the survey. All swine farms in this study were closed-site farms. In total, 26 (52%) swine farms had introduced pigs from January to May 2020. On these farms, the frequency of pig introductions ranged from 1 to 25, with a median of 2. The maximum number of pigs introduced was 15,000 and the minimum was 5 (median = 798.5). Of all the farms, 38 (76%) farms were all-in/all-out, and 12 (24%) were not (Table 1).

### 3.2. Disease Status

Out of 50 valid farms in the survey, 14 of them recorded viral diseases during January to May 2020 and were defined as viral disease positive farms (Figure 1). The overall farm level prevalence of viral disease was 28.0% (95%CI 16.2–42.5%). Seven farms recorded porcine reproductive and respiratory syndrome (PRRS), three recorded porcine epidemic diarrhea (PED), and one recorded pseudorabies (PR). The three farms reported viral disease occurrences but refused to release the disease names. All viral disease were confirmed by reverse transcription polymerase chain reaction (RT-PCR). None of these farms ever recorded an African swine fever (ASF) outbreak since November 2018 when ASF entered China.

### 3.3. Risk Analysis

Detailed percentages of the samples in the different categories of each variable are listed in Table 2, followed by the prevalence of viral diseases, odds ratios, and statistical difference tests results (*p* value). All farms (50/50, 100%) required vehicles to be disinfected before entering the farm; thus, this variable was not listed in Table 2.

There were 78.0% old (established before ASF outbreak) farms and 22.0% new (established after ASF outbreak) farms included in our study, but no statistical difference was found regarding viral disease (PRRS, PED, and PR) positivity. Moreover, most of the farms presented poor biosecurity practices. For example, as high as 70.0% of the farms implemented the all-in-all-out strategy, and only 48.0% of farms applied pathogen testing before introducing any new pigs into the farm. Most of the farms (86.0%) required the workers to change coats and boots before entering the working area. However, as high as 94.0% of farms did not require their workers to wash hands before working.

Univariable analysis selected three variables for multivariable analysis, time point of establishment (*p* = 0.144), the method of dead pig disposal (*p* = 0.018), and the hand-washing requirement (*p* = 0.186). The multivariable logistic regression model indicated that dead pigs collected and disposed of by the others posed a significant high risk (adjusted *p* = 0.016, <0.05) of virus infection to the farms in our survey. The odds of ‘removal by others’ to ‘on-farm incineration pit’ were as high as 8.0 (95%CI 1.5–43.5) (Table 3). The *p* value of the Hosmer and Lemeshow test was equal to 1.000 (>0.05), and the area under ROC (AUC) was 0.695 (>0.6).

## 4. Discussion

From January to May 2020, the Chinese government applied the most restricted movement control measures to fight the COVID-19 pandemic [16]. During the same time, food supply chains, including the farming, processing, and distribution, were required to keep stable supplies to guarantee people’s daily consumption [17]. This requirement was extremely difficult for the pig industry in China, which had been destroyed badly due to ASF outbreaks since November 2018. As of December 2019, a total of 110 outbreaks were reported, and 582,415 pigs had died, either due to this deadly virus or the control of virus spreading [18]. Under the pressures from these two epidemics, the importance of biosecurity practices in intensive pig farms were highlighted. Therefore, we designed this cross-sectional study to unveil the potential factors that posed risks to these farms and to provide evidence-based suggestions to all stakeholders in the pig industry, such as governmental officers and farm owners. Our study covered 12 provinces in China; thus, it should shed some light on the overall state of Chinese intensive pig farms.

Porcine reproductive and respiratory syndrome (PRRS) was the most prevalent virtual disease found in our study, and its first outbreak in 1995 was reported in northern China [19,20]. However, it soon spread to all parts of China in the following three years [20]. In 2006, a highly pathogenic Chinese variant of the PRRS virus started its quick spreading which resulted in a national epidemic and caused 400,000 deaths [21]. Coincidentally with the ASF in China, its pathway started from poor biosecurity in backyard and small farms, via medium farms, and finally ended up with intensive pig farms [20]. Unfortunately, in the 2006 PRRS epidemic, the majority of the research attention was paid to the pathogenic variation and vaccination but not to the epidemiological approaches [19,20,21]. Both porcine epidemic diarrhea (PED) and pseudorabies (PR) have been documented in China for around 50 years [22,23]. Both were initially controlled by vaccination but re-emerged due to their variant strains. All three diseases were enrolled in the “Mid- and Long-term Animal Disease Prevention and Control Program in China (2012–2020)”. The plan of this program was to eradicate priority animal diseases in China by the end of 2020 (The State Council of the People’s Republic of China, 2012) [23]. Until now, these three diseases are still endemic in many provinces in China, but with the improvement of biosecurity measures for preventing ASF, the prevalence of all infectious diseases should be decreasing.

The Chinese government has been encouraging large-scale pig farming since 2017 for the purpose of reducing the environmental pollution from backyard farming [24]. However, before the 2018 ASF outbreak in China, the number of small retail investors was still relatively large. For example in 2016, pig farms raising less than 500 pigs per year accounted for 60% of total pig industry [25]. However, the African swine fever outbreaks had great impacts on these small pig farms in China, accounting for 71.4% of the total number of infected pig farms [25]. This was due to poor biosecurity practices, such as introducing new pigs without quarantine and disease testing, no restrictions on visitors, and no regular disinfection [23]. In addition to small pig farms, some large-scale pig farms also have biosecurity deficiencies and loopholes [26]. However, the ASF outbreak boosted biosecurity levels in large farms together with the COVID-19 pandemic since farm owners found that a high level of biosecurity would protect their farms from ASF infections [27]. During the early stage of lockdown, the introduction of new animals was affected since trucks could not be used and the highway was on lockdown [28]. In the study, we found 100% of the pig farms required disinfection of the vehicles before entering pig farms. Nevertheless, we still found some deficiencies in the biosecurity of these large-scale farms, such as the inability to carry out all-in-all-out operations and not requiring workers to wash their hands. All-in, all-out has always been a highly recommended pig farm management system since it would effectively eliminate the pathogen in the environment and improve pig performance [29,30]. Hand hygiene was always believed to be the most important tool to prevent nosocomial infections [31]. In the current COVID-19 outbreak, hand washing was also advised as the most important measure of COVID-19 prevention and control for individuals to take [32]. Even though these two factors did not show risks in the logistic model which may be due to the small sample size, their importance should not be ignored.

In this study, we found one statistically significant risk factor, ‘disposal of dead pigs’. ‘Removal by others’ was found to be associated with the higher prevalence of viral diseases compared with the “on-farm incineration pit”. This may be due to pig buyers carrying dead pigs collected from different places and moving them between farms, thereby increasing the risk of virus transmission between farms. Li et al. reported that traders and trade workers often mix pigs from different farms to make a batch for transport, which will increase the risk of pigs being infected with different influenza strains [33]. Some countries legally require on-farm burning or burying dead pigs [9]. In China, to upgrade the pig industry and establish a standardized production system, the Chinese government is promoting the transformation, acceleration, and making of policies to subsidize the harmless treatment of diseased and dead livestock, which are conducive to disease prevention and control in pig farms [12]. However, the government prohibits the purchase and sale of dead pigs but does not pointed out a specific way of disposal [12].

Dead pigs collected by others were most likely to be processed into food, such as sausages [34]. We found several cases of collecting sick dead pig and processing them to be manufactured into food. In the end, both dead pig collectors and processors were committing a crime in violation of the Animal Epidemic Prevention Law [35,36]. For example, in Fujian province, police reported 2000 tons of pork were collected from dead pigs and processed to be frozen meat and pot-stewed meat [35]. As another example, in Sichuan province, an outbreak of *Streptococcus suis* in humans caused more than 200 people to be infected and was associated with *Streptococcus suis* in sick dead pigs via direct contact, such as slaughtering and processing dead pigs [36,37]. To extend the preservation, traditional and widely accepted Chinese food was normally processed by drying and salting. Pork products, such as salt-cured meat, was one of the Sichuan people’s favorite foods, which was also a must-eat food in important festivals. The high demand for these manufactured foods was easily used to cover the abnormal color and flavor of the meat from dead pigs. Moreover, virus-carrying meat or meat products, such as ASF-virus-carrying sausage, has a high risk of causing a new outbreak through swill [26]. In summary, requiring on-farm incineration of dead pigs will not only help with reducing virus transmission, but also improve food safety.

In addition, in this study we expected to find out whether daily activities during the lockdown period had an impact on the occurrence of the disease. However, we found that 100% (50/50) of farms did not allow visitors to enter farms. Following the outbreak of African swine fever in China in 2018, all pig farms reduced the frequency of personnel entering and leaving the farm, almost all farms adjusted the entrance to one, and all personnel movements were restricted [27]. Therefore, we just included the factor of whether the farm establishment was before or after the ASF outbreak.

## 5. Conclusions

To conclude, this study demonstrated a high farm-level prevalence (28.0%, 95%CI: 16.3–42.5%) of viral diseases in 50 intensive farms in 12 provinces of China. Intensive farms with dead pigs ‘removal by others’ were found to be significantly associated with viral disease positivity. In addition, adapting the all-in-all-out system and an on-farm incineration pit while also requiring workers to wash their hands regularly, can also reduce the risk of virus transmission, even though this factor did not show statistical significance in our study. The results of our study could help to design better surveillance strategies in China and other countries.

## Figures and Tables

**Table 1:** Names, descriptions, and coding of the 19 variables and their categories included in the study as potential risk factors for viral diseases in the pig farms in China.

Variables	No. of Herds (*n* = 50)
Types of farms	
Commercial grow-to-finish swine farms	3
Commercial farrow-to-finish swine farms	21
Breeding swine farms	19
Wean-to-finish swine farms	7
Year of farm establishment	
Before ASF outbreak	11
After ASF outbreak	39
Total number of pigs in the farm	
>5000	12
2000~5000	17
<2000	21
Breeds in the farm	
Single species	31
Multispecies	19
Farms level all in/all out	
Yes	38
No	12
House level all in/all out	
Yes	35
No	15
Type of environment around the farm	
Village	24
Mountain or hill	13
Highway	13
Did you import any pigs between 1 January 2020 to 31 May 2020 and test these pigs before they were introduced? (Testing before introducing new pigs)	
Yes	26
No	24
If vehicles are required to be disinfected before entering the farm	
Yes	50
No	1
Are visitors allowed to enter the farm	
Yes	0
No	50
Did you sell any pigs between 1 January 2020 to 31 May 2020? (Selling activity from Jan to May 2020)	
Yes	35
No	15
Loading person when selling	
Workers	42
Buyers	4
Both	4
Coat changing before picking pigs	
Yes	43
No	7
Boot changing before picking pigs	
Yes	43
No	7
Hand washing before picking pigs	
Yes	3
No	47
Place of diagnosis	
Resident veterinarian	39
Township veterinary station	2
Services company	3
University	1
Other units	5
Have any animals on your farm ever tested positive for any viral diseases between 1 January 2020 to 31 May 2020?(Disease status in the past six month)	
Yes	14
No	36
Not sure	0
If yes, please write the disease name.	
PRRS 7	
PED 3	
PRRS 1	
I don’t want to release the name. 3	
Which method was used to test your pigs for those diseases?	
RT-PCR	14
Other methods	0
Does the farm have facilities or measures for on-farm harmless disposal?(On-farm harmless disposal)	
Yes	50
No	0
If yes, what facilities or measures were used?	
Removal by others	8
On-farm incineration pit	42

**Table 2:** Univariable analyses of the variables that have potential risks to farm level viral disease positivity.

Variables	Categories	Percentage (%)	Prevalence, 95%CI (%)	Odds Ratio, 95%CI	*p* Value
Time point of establishment					0.144 *
	After 2018 ASF outbreak	22	45.5 (16.7–76.6)	2.8 (0.7–11.3)	
	Before 2018 ASF outbreak	78	23.1 (11.1–39.3)	1.0	
Farm size					0.375
	>5000	24	41.7 (15.2–72.3)	3.0 (0.6–14.8)	
	2000~5000	34	29.4 (10.3–56.0)	1.8 (0.4–8.0)	
	<2000	42	19.0 (5.4–41.9)	1.0	
Number of breeds					0.836
	Only one	62	29.0 (14.2–48.0)	1.1 (0.3–4.1)	
	More than one	38	26.3 (9.1–51.2)	1.0	
Mountain or hill near farm (<3 km)					0.239
	Yes	26	15.4 (1.9–45.4)	0.8 (0.6–1.0)	
	No	74	32.4 (18.0–49.8)	1.0	
Highway near farm (<3 km)					0.201
	Yes	26	15.4 (1.9–45.4)	0.8 (0.6–1.0)	
	No	74	32.4 (18.0–49.8)	1.0	
Testing before introducing new pigs					0.420
	Yes	48	33.3 (15.6–55.3)	1.7 (0.5–5.8)	
	No	52	23.1 (9.0–43.6)	1.0	
Purchasing activity from Jan to May 2020					0.650
	Yes	52	30.8 (14.3–51.8)	1.3 (0.4–4.6)	
	No	48	25.0 (9.8–46.7)	1.0	
Selling activity from Jan to May 2020					0.409
	Yes	70	31.4 (16.9–49.3)	1.8 (0.4–7.8)	
	No	30	20.0 (4.3–48.1)	1.0	
House level all-in-all-out strategy					0.891
	No	30	26.7 (7.8–55.1)	1.0	
	Yes	70	28.6 (14.6–46.3)	1.1 (0.3–4.3)	
Disposal of dead pigs					0.018 *
	Removal by others	16	62.5 (24.5–91.5)	6.1 (1.2–30.3)	
	On-farm incineration pit	84	21.4 (10.3–36.8)	1.0	
Coat changing requirement for workers					0.384
	Yes	86	30.2 (17.2–46.1)	2.6 (0.3–23.8)	
	No	14	14.3 (0.4–57.9)	1.0	
Boot-changing requirement for workers					0.384
	Yes	86	30.2 (17.2–46.1)	2.6 (0.3–23.8)	
	No	14	14.3 (0.4–57.9)	1.0	
Hand-washing requirement for workers					0.186 *
	Yes	6	66.7 (9.4–99.2)	5.8 (0.5–70.2)	
	No	94	25.5 (13.9–40.3)	1.0	

* Variables with *p* < 0.20 in the univariable analysis. They were kept to enter into the multivariable logistic model.

**Table 3:** Multivariable logistic regression model for potential risk factors selected from univariable analyses.

	β	Odds Ratio, 95%CI		*p* Value
Farm established before 2018 ASF outbreak in China	−1.1	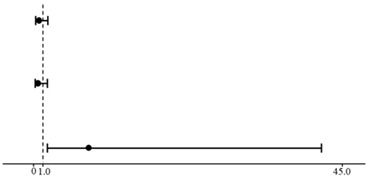	0.3 (0.1–1.6)	0.178
No hand-washing requirement for workers	−2.2		0.1 (0.0–1.5)	0.100
Dead pig removal by others	2.1		8.0 (1.5–43.5)	0.016
Constant	1.5			

Note: The chart in the middle of the table was used to visualize the level of risk of each variable compared with the reference value of 1.0. Dots on the chart indicate the value of the odds ratio, and the interval bars represented the 95%CI of the odds ratio.

## Data Availability

Not applicable.
